# Pregnancy Termination Among Women of Reproductive Age: Evidence from the Indonesian Demographic and Health Survey

**DOI:** 10.3390/ijerph22040564

**Published:** 2025-04-04

**Authors:** Rosnani Rosnani, Rifky Octavia Pradipta, Bayu Satria Wiratama, Nelsensius Klau Fauk, Paul Russell Ward, Heri Kuswanto, Nikson Sitorus, Joni Haryanto, Hidayat Arifin

**Affiliations:** 1 Department of Maternity, School of Nursing, Politeknik Kesehatan Kemenkes Palembang, Palembang 30135, Indonesia; 2 Department of Basic Nursing, Faculty of Nursing, Universitas Airlangga, Surabaya 60115, Indonesia; rifky-op@fkp.unair.ac.id (R.O.P.); joni-h@fkp.unair.ac.id (J.H.); hidayat.arifin@fkp.unair.ac.id (H.A.); 3 Research Group in Medical-Surgical Nursing, Faculty of Nursing, Universitas Airlangga, Surabaya 60115, Indonesia; 4 Department of Biostatistics, Epidemiology, and Population Health, Faculty of Medicine, Public Health, and Nursing, Universitas Gadjah Mada, Yogyakarta 55281, Indonesia; bayu.satria@ugm.ac.id; 5 Centre for Public Health, Equity and Human Flourishing, Torrens University Australia, Adelaide, SA 5000, Australia; paul.ward@torrens.edu.au; 6 Department of Statistics, Institut Teknologi Sepuluh Nopember, Surabaya 60111, Indonesia; heri.kuswanto@its.ac.id; 7 Research Center for Public Health and Nutrition, National Research and Innovation Agency, Jakarta 10340, Indonesia; nikson.sitorus@brin.go.id

**Keywords:** pregnancy termination, abortion, reproductive age, women, Indonesia, Demographic and Health Survey

## Abstract

The consequences associated with pregnancy termination have garnered attention from healthcare professionals, particularly in Indonesia. However, national-level evidence on the factors driving pregnancy termination in Indonesia remains limited. This research aimed to identify patterns and characteristics associated with pregnancy termination among reproductive-age women in Indonesia. A cross-sectional study analyzed secondary data from the 2012 and 2017 Indonesian Demographic and Health Survey, involving women aged 15–49. The weighted sample included 42,269 individuals in 2012 and 47,001 in 2017. Binary logistic regression identified the correlates of pregnancy termination. Among 89,270 women of reproductive age, the prevalence of pregnancy termination was 12.68% in 2012 and 12.95% in 2017. Pregnancy termination was more frequently reported among women aged 44–49 years (adjusted odds ratio (AOR): 4.34, 95% confidence interval (CI): 3.54–5.33), those with secondary education (AOR: 1.29, 95% CI: 1.14–1.46), married women (AOR: 195.40, 95% CI: 114.70–332.90), employed women (AOR: 1.05, 95% CI: 1.00–1.09), individuals with health insurance (AOR: 1.07, 95% CI: 1.02–1.11), those who had experienced domestic violence (AOR: 1.07, 95% CI: 1.02–1.11), and regular television viewers (AOR: 1.10, 95% CI: 1.05–1.15). Conversely, pregnancy termination was less commonly reported among women with 1–2 living children (AOR: 0.80, 95% CI: 0.74–0.87), those who expressed no preference for having more children (AOR: 0.89, 95% CI: 0.84–0.94), and women using modern contraception (AOR: 0.76, 95% CI: 0.72–0.80). The findings revealed that the prevalence did not observe any changes in the incidence of pregnancy terminations between 2012 and 2017. Further evaluation by healthcare professionals is crucial to understanding the reasons behind pregnancy termination, especially among women of reproductive age. Insights into factors related to pregnancy termination, especially sociodemographic factors, can help mitigate the pregnancy termination in this population.

## 1. Introduction

Pregnancy termination continues to be a significant health issue facing many countries globally, and Indonesia is not an exception. It refers to the intentional ending of a pregnancy through medical or surgical methods before fetal viability, encompassing induced abortions, miscarriages, stillbirths, and medically advised terminations for health reasons [1,2]. Between 2015 and 2019, there were an estimated 121 million unintended pregnancies annually worldwide, translating to a prevalence of 64 unintended pregnancies per 1000 women aged 15–49 years. Among these 121 million unintended pregnancies, 61% ended in abortion, amounting to approximately 73 million abortions, or 39 abortions per 1000 women in the same age group [3]. This high rate of unintended pregnancies and terminations reflects a significant public health concern. The decision to undergo an abortion among women of reproductive age is influenced by a complex interplay of socioeconomic, educational, marital, and health service factors [4,5], making it imperative to understand the underlying risk factors of pregnancy termination to address this issue effectively.

Factors influencing pregnancy termination or abortion are diverse and context-specific. In low–middle-income countries (LMICs), being wed, having more offspring, and being older are strongly associated with a higher likelihood of pregnancy termination [6]. Having secondary or higher education raises the probability of abortion across multiple studies, while uneducated women and those in rural areas are less likely to terminate pregnancies [7,8,9]. Other significant contributors to pregnancy termination include the desire to prevent further childbearing after achieving a specific number of children, contraceptive use, exposure to intimate partner violence, and employment status [9,10,11]. Among younger women, particularly those in their twenties, work-related factors and unstable relationships often influence their decision to terminate a pregnancy, while older women are more likely to be influenced by partner instability or professional commitments [10,12]. Additionally, socioeconomic factors such as wealth, access to contraceptive information, and geographic region also play a role in differentiating the likelihood and safety of abortions [13].

In the context of Indonesia, although pregnancy termination has not received much academic attention, the problem has been addressed at the policy level and is encapsulated in Government Regulation Number 61 of 2014. This regulation legalizes abortion under specific circumstances, such as pregnancies resulting from rape that may cause psychological trauma. It aims to protect the reproductive rights of rape victims, aligning with the human rights principles [14]. Moreover, the Indonesian welfare state model, encompassing healthcare programs, obligates the government to promote the welfare of its citizens and improve reproductive health policies [15]. The Indonesian government has implemented initiatives to reduce pregnancy termination rates by focusing on reproductive health education, family planning services, and maternal health care [16]. The National Family Planning Program promotes contraceptive use [16,17,18], while the Maternal and Child Health Program improves prenatal and postnatal care [17,19]. Reproductive health education in schools and communities provides young people with information about safe sex practices [18]. These efforts aim to reduce unintended pregnancies, enhance maternal health, and decrease the need for pregnancy termination in Indonesia.

Several studies have explored specific aspects of pregnancy termination in Indonesia. Lubis et al. [20] analyzed the issue of unwanted pregnancy, Ermiati et al. [21] assessed the decision-making process surrounding unwanted pregnancy, and Musoddaq et al. [22] identified factors associated with miscarriages among women of reproductive age. These studies analyzed a single dataset only and none reported on factors associated with pregnancy termination or induced abortion among women of reproductive age in Indonesia, which are addressed through this study. The study aims to fill in this knowledge gap and provide comprehensive information by evaluating the determinants of pregnancy termination among women of reproductive age in Indonesia between 2012 and 2017. This study is the first to evaluate the national prevalence of pregnancy termination in Indonesia using DHS data from 2012 and 2017. It examines various contributing factors to pregnancy termination, including demographics, socioeconomic status, reproductive preferences, media exposure to family planning, and domestic violence. It also provides a comparison of national data in the years 2012 and 2017. This comparison is facilitated by the suitability of variables and data that were available in Indonesia during this period. Specifically, this study seeks to answer the following questions: (1) What is the prevalence of pregnancy termination among reproductive-age women in Indonesia? (2) What are the characteristics and patterns observed among women who have experienced pregnancy termination in Indonesia? Understanding these factors can be useful to inform the development of evidence-based interventions that address the needs of pregnant women and prevent pregnancy termination.

## 2. Materials and Methods

### 2.1. Study Design

A cross-sectional examination was designed for this current study using 2012 and 2017 datasets of the Indonesian Demographic and Health Survey (IDHS) [1]. This survey was funded and provided by the Indonesian government and covered all 34 provinces of Indonesia. Technical support for the survey was provided by ICF International through The DHS Program.

### 2.2. Data Sources and Samples

The 2012 and 2017 IDHS were comprehensive national surveys conducted by Statistics Indonesia, in collaboration with the National Population and Family Planning Board and the Ministry of Health. The 2012 survey involved approximately 45,607 women, interviewing an equal number of women aged 15–49. In 2017, the survey expanded to include about 49,627 households, with interviews conducted with 49,627 women aged 15–49. Both surveys utilized a stratified, multistage cluster sampling method to ensure nationally representative data. This method involved dividing the population into census blocks, with each block consisting of a fixed number of households. In both the 2012 and 2017 surveys, each census block included 25 households. This approach ensured that the data collected accurately represented the diverse regions and populations across Indonesia.

This study focuses on women of reproductive age, classified by WHO as ranging from 15 to 49 years [23]. Weighting was based on the number of provinces in Indonesia to ensure that the survey results were accurate, reliable, and truly reflective of the entire population. The 2012 IDHS comprised 45,607 observations with 4167 variables, from which about 3338 observations were excluded due to missing data. In the 2017 IDHS, 49,627 observations and 5491 variables were recorded, but approximately 2626 observations were omitted due to missing values. Consequently, the study included 42,269 observations from the 2012 IDHS and 47,001 from the 2017 IDHS.

### 2.3. Measurements

The dependent variable in this study was pregnancy termination (ever had a terminated pregnancy). In the context of the Demographic and Health Surveys (DHSs), “Ever had a pregnancy terminated” refers to whether a woman has ever experienced the end of a pregnancy before the fetus was viable, either through miscarriage (spontaneous abortion), induced abortion (intentional termination), or stillbirth [2]. The specific question to evaluate the pregnancy termination is “Have you ever had a pregnancy that miscarried, was aborted, or ended in a stillbirth?” The independent variables included demographic characteristics (age, education levels, marital status, residence, and current living children), socioeconomic (employment status, wealth index, and health insurance), reproductive preference (fertility preferences, and current contraceptive used), media exposure about family planning (radio, television, and newspaper), and other factors such as domestic violence.

In the demographic characteristics, women of reproductive age were classified into seven categories, each spanning five years (15–19, 20–24, 25–29, 30–34, 35–39, 40–44, and 45–49). Educational levels were classified into four categories (no education, primary, secondary, and higher). Marital status was classified into two categories (never married and married). The place of residence was categorized as “urban” or “rural”. Current living children was categorized into “none”, “1–2”, “3–5”, and “6 or more”. In the socioeconomic factors, employment status was categorized into “no” and “yes”. The wealth index, determined based on principal component analysis, was categorized from “poorest” to “richest”. Health insurance was categorized into “no” and “yes”. According to reproductive preference, fertility preference was categorized into “have another”, “undecided”, and “no more”. The current contraceptive use was categorized into “not using”, “modern”, and “traditional”. The study also evaluated media exposure to family planning from radio, television, and newspapers, with these three variables categorized into “no” and “yes”. Another factor evaluated was domestic violence, with variables such as “beating if refuses to have sex with a partner”, “beating if argues with a partner”, and “beating if goes out without telling partner”; these were then categorized as “no” and “yes”.

### 2.4. Statistical Analysis

This study followed the guidelines from Strengthening the Reporting of Observational Studies in Epidemiology (STROBE), which are designed to improve the reporting of observational studies. STATA version 16.1 software (Stata Corp., College Station, TX, USA) was used to process the analysis. Descriptive statistics for demographic characteristics were computed and summarized in Table 1. At the bivariate analysis level, the Chi-Square test was used to evaluate the relationship between independent and dependent variables. Variables with a *p* value ≥ 0.25 were not included in further analysis. Binary logistic regression was used at the multivariate analysis level to determine factors contributing to pregnancy termination. Weighted percentages were presented to achieve proportional results. Overall, three models were computed in this study. Model 1 analyzed the characteristics and patterns of women who reported pregnancy termination in 2012, while Model 2 examined these characteristics in 2017. Model 3 explored these patterns across both 2012 and 2017 (combined). Combining the 2012 and 2017 IDHS data enhances statistical power, enables trend analysis, and provides a more comprehensive understanding of pregnancy termination patterns over time. In our findings, we reported the adjusted odds ratio (AOR) along with a 95% confidence interval (CI) and a *p* value of less than 0.05. To account for the clustering effects and sampling weight resulting from the multistage cluster random sampling used in data collection, we utilized the “svy” survey commands in STATA, as this was a national survey.

## 3. Results

### 3.1. Descriptive Statistics of Participant Characteristics

A total of 42,269 participants from the 2012 Indonesian Demographic and Health Survey (IDHS) and 47,001 participants from the 2017 IDHS were included in this study. The study observed that pregnancy termination prevalence did not observe any changes from 12.7% (95% CI: 12.20–13.18) in 2012 to 13.0% (95% CI: 12.54–13.37) in 2017. There was a notable increase in educational attainment among participants, with the percentage of those with secondary education rising from 51.2% in 2012 to 55.2% in 2017 and the proportion of those with higher education increasing from 12.5% in 2012 to 15.8% in 2017. In terms of marital status, the proportion of participants who had never married increased from 25.3% in 2012 to 27.0% in 2017, indicating a slight decrease in the number of married participants. The percentage of participants living in urban areas decreased from 52.8% in 2012 to 51.8% in 2017. There was an approximate 1% increase in the number of nulliparous participants from 2012 to 2017. The study also found an increase in the number of unemployed women, from 44.3% in 2012 to 46.3% in 2017, while the wealth index showed little change over the same period. Health insurance ownership saw a significant increase, rising by 20% from 2012 to 2017. Additionally, there was an increase in the desire among participants to have more children and to use traditional contraception in 2017 compared to 2012. The dissemination of information related to family planning via radio and newspapers decreased, while television remained a dominant source in 2017. Finally, the incidence of domestic violence decreased by 6% from 2012 to 2017. These findings highlight several important trends and changes in the Indonesian population between 2012 and 2017 (Table 1).

### 3.2. Association Between Participant Characteristics and Pregnancy Termination

In 2012 and 2017, pregnancy termination was more frequently reported among women with varying characteristics, including age, education level, marital status, current living children, employment status, health insurance, fertility preference, contraceptive use, and experience of domestic violence (*p* < 0.05). Additionally, in 2012, differences were also observed based on residence and wealth index (*p* < 0.05). In the combined analysis, pregnancy termination patterns varied by age, education level, marital status, residence, current living children, employment status, health insurance, fertility preference, contraceptive use, and media exposure (radio and television), as well as experience of domestic violence (*p* < 0.05) (Table 2).

### 3.3. Predictors of Pregnancy Termination Among Women of Reproductive Age

In the adjusted analysis of Model 1, pregnancy termination was more frequently reported among women with specific characteristics, including age, education level, marital status, current living children, wealth index, health insurance, fertility preference, contraceptive use, radio exposure, and experience of domestic violence. An increase in age was associated with higher odds of reporting pregnancy termination. Women aged 45–49 years (AOR: 4.29, 95% CI: 3.26–5.64) were about four times more likely to have experienced pregnancy termination compared to younger women aged 15–19 years. Women with secondary education (AOR: 1.50, 95% CI: 1.26–1.78) were approximately one and a half times more likely to report pregnancy termination compared to women with no education. Married women were more frequently found in the group reporting pregnancy termination, with an AOR of 4.28 (95% CI: 3.71–4.93) compared to those who had never married. Regarding the number of living children, women with 1–2 living children (AOR: 0.76, 95% CI: 0.68–0.86) were less likely to report pregnancy termination compared to those with no children. Women in the richer wealth category (AOR: 0.89, 95% CI: 0.80–0.98) had lower odds of reporting pregnancy termination than those in the poorest wealth category. Additionally, women with health insurance (AOR: 1.08, 95% CI: 1.02–1.15) were more likely to report pregnancy termination compared to those without health insurance. Women who expressed no preference for having more children (AOR: 0.90, 95% CI: 0.83–0.98) and those using modern contraception (AOR: 0.74, 95% CI: 0.70–0.80) were less likely to report pregnancy termination compared to their counterparts. Exposure to family planning information via radio was associated with higher odds of reporting pregnancy termination (AOR: 1.11, 95% CI: 1.01–1.23) compared to those not exposed. Furthermore, women who had experienced domestic violence (AOR: 1.11, 95% CI: 1.04–1.19) were more likely to report pregnancy termination than those who had not experienced domestic violence (Table 3).

In the adjusted analysis of Model 2, pregnancy termination was more frequently reported among women with specific characteristics, including age, marital status, number of living children, fertility preference, contraceptive use, and experience of domestic violence. Women aged 40–44 years (AOR: 4.48, 95% CI: 3.28–6.12) were approximately four times more likely to report pregnancy termination compared to younger women. Married women (AOR: 3.66, 95% CI: 3.20–4.17) were more frequently found in the group reporting pregnancy termination compared to those who had never married. Similar to Model 1, women with 1–2 living children (AOR: 0.84, 95% CI: 0.74–0.94) were less likely to report pregnancy termination compared to those with no living children. Women who expressed no preference for having more children (AOR: 0.88, 95% CI: 0.82–0.95) and those using modern contraception (AOR: 0.78, 95% CI: 0.73–0.84) were also less likely to report pregnancy termination compared to their counterparts. Additionally, women who had experienced domestic violence (AOR: 1.14, 95% CI: 1.07–1.22) were more likely to report pregnancy termination than those who had not experienced domestic violence (Table 3).

In the adjusted analysis of Model 3, pregnancy termination was more frequently reported among women with specific characteristics, including age, education level, marital status, number of living children, employment status, health insurance, fertility preference, contraceptive use, television exposure, and experience of domestic violence. Older women aged 45–49 years (AOR: 4.34; 95% CI: 3.54–5.33) were approximately four times more likely to report pregnancy termination compared to younger women. Women with secondary education were more frequently found in the group reporting pregnancy termination compared to those with no education (AOR: 4.34; 95% CI: 3.54–5.33). Married women (AOR: 3.95, 95% CI: 3.59–4.36) were also more likely to report pregnancy termination compared to those who had never married. Regarding the number of living children, women with 1–2 living children (AOR: 0.80, 95% CI: 0.74–0.87) were less likely to report pregnancy termination compared to those with no living children. Working women (AOR: 1.05, 95% CI: 1.00–1.09) and those with health insurance (AOR: 1.07, 95% CI: 1.02–1.11) were more frequently found among those reporting pregnancy termination compared to their counterparts. Consistent with the previous models, women who expressed no preference for having more children (AOR: 0.89, 95% CI: 0.84–0.94) and those using modern contraception (AOR: 0.76, 95% CI: 0.72–0.80) were less likely to report pregnancy termination compared to their counterparts. Exposure to family planning information via television was associated with a higher likelihood of reporting pregnancy termination (AOR: 1.10, 95% CI: 1.05–1.15) compared to those without such exposure. Additionally, women who had experienced domestic violence (AOR: 1.13, 95% CI: 1.05–1.15) were more likely to report pregnancy termination than those who had not experienced domestic violence (Table 3).

## 4. Discussion

The termination of pregnancy in women of reproductive age, which occurs when a fetus is not viable and is terminated either through miscarriage, induced abortion, or stillbirth, is a matter of great healthcare concern due to its impact on maternal mortality. This study examines the prevalence of pregnancy termination in women of reproductive age in Indonesia and the associated factors using the 2012–2017 DHS data. The findings of a study conducted in 2007 revealed that 12.68% of women had undergone pregnancy termination, which rose to 12.95% in 2017. This prevalence is higher than that of countries such as Sierra Leone, Mozambique, and Ethiopia, where it consistently remained around 9% [24,25,26]. While differences between countries could be due to study periods, target populations, and accessibility to healthcare facilities, the high prevalence in Indonesia is a cause for concern and warrants attention from healthcare workers and the Indonesian government.

Indonesia, as the fourth most populous country in the world, offers valuable insights into reproductive health trends in a large and diverse population [27]. Understanding pregnancy termination patterns in Indonesia provides an opportunity to assess the impact of sociodemographic and policy-related factors in a setting where abortion laws are restrictive [14], yet unintended pregnancies remain a major public health concern. Given that similar restrictive policies exist in many LMICs, findings from Indonesia can inform international reproductive health policies, particularly in countries facing comparable sociocultural factors. By comparing Indonesia’s situation with data from other nations, this study contributes to global discussions on the determinants of pregnancy termination. Previous studies have examined pregnancy termination using Demographic and Health Survey (DHS) datasets from various countries, such as Ethiopia, Mozambique, and Ghana [24,25,26]. This study follows a similar approach by analyzing the Indonesian DHS, allowing for comparisons with other LMICs and contributing to a broader understanding of pregnancy termination trends in different sociocultural contexts. The study’s findings not only provide country-specific recommendations but also offer a comparative perspective that can enhance reproductive health policies on a global scale.

This study identified a pattern where pregnancy termination was more frequently reported among older women of reproductive age. For instance, women aged 45–49 years had a 4.34 times higher probability to report experiencing pregnancy termination compared to younger women. These results align with prior studies conducted in Sierra Leone, Mozambique, and Ethiopia [24,25,26]. This trend may be due to medical factors, as older women face higher risk of maternal mortality, leading to medically indicated pregnancy termination. The literature shows that advanced maternal age (AMA), defined as age 35 years or older, is associated with an increased risk of maternal and adverse pregnancy [28,29,30,31]. This risk is attributed to a higher prevalence of pre-existing medical comorbidities in older women and the physiological changes associated with aging, which can complicate pregnancy and childbirth [28,31,32]. In addition, it should be noted that within the Indonesian context abortion is only permitted in medical emergencies and rape cases to protect women’s reproductive rights. These laws balance the protection of unborn life with the rights of women facing health risks or sexual violence [14]. However, findings in other settings have suggested that unwanted pregnancies in families with a high number of children can lead to the decision for pregnancy termination due to concern about providing for additional members and the impact on existing family dynamics and resources [33].

This study has suggested that women who have completed their primary and secondary education are more likely to report pregnancy termination than those who are not educated. This trend is particularly pronounced among women who have completed their higher education, aligning with previous findings from studies [24,26]. Educated women may have greater awareness of reproductive rights, access to healthcare services, and family planning options [34,35]. Additionally, they might prioritize [36]. Moreover, their increased financial independence and access to contraceptives may play a role in their decisions to delay or forgo childbearing.

In terms of marital status, unmarried women were less likely to report experiencing pregnancy termination compared to those with other marital statuses, which is consistent with previous findings in other settings [24,26,37]. This may be due to Indonesian regulations that allow only married women to terminate pregnancies for specific health-related reasons [14]. Additionally, unmarried or divorced women are less likely to report experiencing pregnancy termination due to social stigma and perceptions of pregnancy outside marriage as deviant from sociocultural norms within communities in Indonesia [38]. However, further investigation is necessary to understand why never married women have a lower likelihood of abortion. It is possible that this group of women may be ashamed or unwilling to report their data due to social sanctions. This is a critical issue for Indonesian school-aged females, who may seek clandestine abortions due to various factors such as stigma, lack of access to safe abortion services, and laws [39,40]. Therefore, additional research is warranted to better understand these issues.

According to employment status, women who are employed are significantly associated with pregnancy termination. This finding is similar to the prior study [24]; working women may experience higher rates of adverse obstetric outcomes, such as miscarriage and threatened abortion, which could lead to a decision to terminate a pregnancy [41]. Additionally, the stress associated with balancing work and potential motherhood may contribute to this increased likelihood. Employment in certain industries has been associated with higher adjusted odds ratios for miscarriage, indicating that occupational factors may play a role in the decision to terminate a pregnancy [41]. Furthermore, access to health insurance is closely connected with the decision to terminate a pregnancy. This may be due to the fact that specific medical procedures, including those performed for medical reasons, are covered by health insurance, which makes it more convenient for women to undergo pregnancy termination. In Indonesia, insurance coverage extends to abortions that pose a threat to the life of the mother or when the fetus has a severe and incurable condition, such as cardiovascular health being crucial for a healthy pregnancy and postpartum period [42]

In this study, it was discovered that exposure to media coverage about family planning is substantially associated with pregnancy termination. This result is consistent with studies conducted in Sierra Leone, Ethiopia, and Mozambique [24,26,37]. In particular, exposure to television media was associated with a higher likelihood to terminate a pregnancy. This could be attributed to the information about abortion care that is broadcast through television. Television could potentially play a role in either reinforcing stigma associated with abortion or in providing educational content that could lead to more nuanced and informed public views [43,44]. Given the influence of television on public opinion, it is plausible that the way abortion care information is presented on TV could significantly affect societal attitudes towards abortion services [45]. Additionally, women who have access to mass media may have a better understanding of abortion laws and abortion pills [46,47].

Domestic violence is significantly associated with pregnancy termination, as women who have experienced domestic violence are more likely to report having undergone pregnancy termination. This finding aligns with a previous study conducted in Indonesia, which assessed the reasons behind unwanted pregnancy termination [21]. The 2017 study in Indonesia found that 25.5% of reproductive-age women had experienced domestic violence from their partners or husbands [21]. The available evidence indicates that domestic violence increases the likelihood of reporting pregnancy termination. Reducing domestic violence could potentially lead to a decrease in unintended pregnancies and abortions, which would improve maternal and reproductive health outcomes [48]. Addressing domestic violence is not only essential for the immediate safety of individuals but also has broader implications for reproductive health and pregnancy outcomes.

The current findings show that women who have 1–2 children were less likely to report experiencing pregnancy termination compared to those who have no children, which support the previous findings [24,25]. This trend reflects the gradual shift towards the preference for having 1–2 children among married couples over time. Furthermore, contemporary couples who prioritize family size preferences and reproductive behavior tend to believe that a smaller number of children can boost productivity more effectively than a larger number [49]. Moreover, a larger family size typically leads to increased demand within the household, particularly for food and basic necessities [50].

The relationship between fertility preference and pregnancy termination has been demonstrated to be significant. It has been found that individuals who do not wish to have additional children are less likely to undergo pregnancy termination than those who desire more offspring. Furthermore, the utilization of modern contraceptives has been shown to decrease the likelihood of pregnancy termination in women compared to those who do not use contraceptives. A decrease in fertility preferences generally implies a desire for fewer children, which could lead to a use of contraception and potentially a decrease in the number of unintended pregnancies, thereby reducing the need for pregnancy terminations [51,52]. A decrease in fertility preferences is associated with increased contraceptive use; this does not always translate to a decrease in pregnancy terminations. In settings where access to quality family planning services is limited, or where contraceptive use is low due to cultural or personal reasons, unintended pregnancies may still occur at high rates, leading to a sustained or even increased demand for pregnancy termination [53,54].

### 4.1. Implications of the Findings in This Study

The outcomes of this study have implications for policymaking on pregnancy termination in Indonesia, the development of programs to reduce the occurrence of undesired pregnancy termination, and further research. About policy in Indonesia, this study provides nationally representative data that can serve as a basis for formulating additional policies and creating suitable counseling to prevent pregnancy termination. Moreover, initiatives related to reproductive health should begin from a young age to prevent unwanted pregnancies and decrease the rate of pregnancy termination. Collaboration across sectors is crucial, involving academic institutions, communities, healthcare providers, and policymakers. Additionally, evaluating the content of promotional materials disseminated via television is necessary to prevent misunderstandings that could increase the pregnancy termination rate. In this digital age, the Indonesian government can also leverage social media platforms and the internet to disseminate information on the prevention of pregnancy termination. Indonesia is home to diverse religions, cultures, and beliefs, which can be further explored to examine pregnancy termination issues using a local approach.

### 4.2. Strengths and Limitations

The strengths of this research are its extensive national scope and the application of international rules, which contribute to the applicability of the results to the respective population. As the first extensive study in Indonesia, it conducts comprehensive analysis of pregnancy termination from 2012 to 2017. However, the research has certain limitations. The cross-sectional design of the IDHS survey employed in the study limits the ability to establish specific causality of pregnancy termination. There are no specific questions to distinguish between miscarriage, abortion, or stillbirth. Thus, we are unable to present the distribution of these outcomes separately, as the DHS combines all three under the term pregnancy termination. Future research should aim to differentiate between these types of terminations to facilitate more targeted policy recommendations. In addition, IDHS does not provide a specific timeframe for pregnancy termination occurrences. The survey only records whether a respondent has ever experienced a pregnancy termination but does not indicate when it happened. Moreover, the data gathered depends on the self-reported information from the questionnaires, which could introduce potential bias.

## 5. Conclusions

Pregnancy termination among women of reproductive age in Indonesia is an ongoing concern. The study did not observe any changes in the incidence of pregnancy terminations with consistent prevalence rates ranged 12–13% reported from 2012 to 2017. This persistent trend highlights the persistent level of termination incidents over the years studied. Various factors, including demographic characteristics, socioeconomic status, reproductive preferences, and media exposure on family planning, have been identified as significant influences on these rates. To decrease pregnancy termination rates, targeted policies and healthcare initiatives that address these factors must be implemented. This could involve enhancing reproductive health education, increasing access to contraception, and ensuring comprehensive healthcare services that support informed decision-making. By tackling these underlying factors comprehensively, healthcare providers can contribute to improving maternal health outcomes and effectively reducing unintended pregnancies in Indonesia.

## Figures and Tables

**Table 1:** Participant’s characteristics.

Characteristics	2012 (*n* = 42,269) *n* (Weighted %)	2017 (*n* = 47,001) *n* (Weighted %)	2012 and 2017 (*n* = 89,270) *n* (Weighted %)
Pregnancy termination			
No	36,787 (87.32)	40,776 (87.05)	77,563 (87.18)
Yes	5482 (12.68)	6225 (12.95)	11,707 (12.82)
Demographics			
Age			
15–19	6189 (14.16)	6971 (14.25)	13,160 (14.20)
20–24	6089 (13.85)	6483 (13.54)	12,572 (13.69)
25–29	6718 (15.42)	6536 (13.60)	13,254 (14.46)
30–34	6572 (15.32)	6936 (14.63)	13,508 (14.96)
35–39	6375 (15.28)	7312 (16.00)	13,687 (15.66)
40–44	5585 (13.99)	6728 (14.42)	12,313 (14.22)
45–49	4741 (11.98)	6035 (13.57)	10,776 (12.81)
Education levels			
No education	1402 (3.13)	818 (1.59)	2220 (2.32)
Primary education	12,708 (33.20)	11,655 (27.33)	24,363 (30.11)
Secondary education	21,937 (51.16)	25,757 (55.24)	47,694 (53.31)
Higher education	6222 (12.52)	8771 (15.84)	14,993 (14.27)
Marital status			
Never married	11,295 (25.31)	13,655 (26.97)	24,950 (26.18)
Married	30,974 (74.69)	33,346 (73.03)	64,320 (73.82)
Residence			
Urban	21,513 (52.77)	25,223 (51.76)	46,736 (52.24)
Rural	20,756 (47.23)	21,778 (48.24)	42,534 (47.76)
Current Living Children			
None	12,069 (27.01)	14,083 (28.16)	26,152 (27.62)
1–2	18,704 (47.98)	20,747 (48.89)	39,451 (48.46)
3–5	10,377 (22.86)	11,219 (21.57)	21,596 (22.18)
6 or more	1119 (2.15)	952 (1.38)	2071 (1.74)
Socioeconomic			
Employment status			
No	18,577 (44.27)	21,444 (46.30)	40,021 (45.34)
Yes	23,692 (55.73)	25,557 (53.70)	49,249 (54.66)
Wealth index			
Poorest	9588 (16.58)	10,243 (16.75)	19,831 (16 67)
Poorer	8457 (19.11)	8907 (19.06)	17,364 (19 08)
Middle	8085 (20.31)	8990 (20.38)	17,075 (20 34)
Richer	7939 (21.45)	9258 (21.45)	17,197 (21 45)
Richest	8200 (22.55)	9603 (22.36)	17,803 (22 45)
Health insurance			
No	24,914 (62.58)	18,077 (41.66)	42,991 (51.56)
Yes	17,355 (37.42)	28,924 (58.34)	46,279 (48.44)
Reproductive preference			
Fertility preference			
Have another	22,324 (51.37)	25,658 (52.86)	47,982 (52.15)
Undecided	2875 (6.18)	2187 (4.33)	5062 (5.21)
No more	17,070 (42.45)	19,156 (42.81)	36,226 (42.64)
Contraceptive			
Not Using	23,522 (53.21)	26,599 (53.19)	50,121 (53.20)
Modern	17,326 (43.74)	18,074 (42.08)	35,400 (42.87)
Traditional	1421 (3.05)	2328 (4.73)	3749 (3.94)
Media exposure about family planning			
Radio			
No	37,939 (89.73)	42,870 (91.11)	80,809 (90.46)
Yes	4330 (10.27)	4131 (8.89)	8461 (9.54)
Television			
No	23,139 (53.34)	21,776 (43.58)	44,915 (48.20)
Yes	19,130 (46.66)	25,225 (56.42)	44,355 (51.80)
Newspaper			
No	35,746 (84.99)	41,175 (87.99)	76,921 (86.57)
Yes	6523 (15.01)	5826 (12.01)	12,349 (13.43)
Others			
Domestic violence			
No	30,106 (73.58)	35,773 (79.22)	65,879 (76.56)
Yes	12,163 (26.42)	11,228 (20.78)	23,391 (23.44)

**Table 2:** Cross-tabulation of participant characteristics and pregnancy termination among women of reproductive age in Indonesia.

Characteristics	Ever Had a Pregnancy Terminated								
	2012 IDHS (n = 42,269)			2017 IDHS (n = 47,001)			2012 and 2017 IDHS (n = 89,270)		
	No n (Weighted %)	Yes n (Weighted %)	*Χ*^2^ *(P)*	No n (Weighted %)	Yes n (Weighted %)	*Χ*^2^ *(P)*	No n (Weighted %)	Yes n (Weighted %)	*Χ*^2^ *(P)*
Demographics									
Age			2300 (<0.001)			3100 (<0.001)			5400 (<0.001)
15–19	6120 (99.14)	69 (0.86)		6923 (99.44)	48 (0.56)		13,043 (99.30)	117 (0.70)	
20–24	5755 (94.72)	334 (5.28)		6177 (95.27)	306 (4.73)		11,932 (95.00)	640 (5.00)	
25–29	6015 (90.24)	703 (9.76)		5915 (90.66)	621 (9.34)		11,930 (90.45)	1324 (9.55)	
30–34	5525 (85.07)	1047 (14.93)		5882 (85.85)	1054 (14.15)		11,407 (85.47)	2101 (14.53)	
35–39	5175 (81.63)	1200 (18.37)		5899 (81.43)	1413 (18.57)		11,074 (81.52)	2613 (18.48)	
40–44	4458 (79.86)	1127 (20.14)		5269 (78.85)	1459 (21.15)		9727 (79.32)	2586 (20.68)	
45–49	3739 (79.85)	1002 (20.15)		4711 (78.87)	1324 (21.13)		8450 (79.30)	2326 (20.70)	
Education levels			205.78 (<0.001)			252.35 (<0.001)			445.31 (<0.001)
No education	1212 (86.93)	190 (13.07)		673 (82.69)	145 (17.31)		1885 (85.40)	335 (14.60)	
Primary education	10,610 (83.38)	2098 (16.62)		9642 (83.54)	2013 (16.46)		20,252 (83.45)	4111 (16.55)	
Secondary education	19,398 (89.50)	2539 (10.50)		22,607 (88.22)	3150 (11.78)		42,005 (88.80)	5689 (11.20)	
Higher education	5567 (88.93)	655 (11.07)		7854 (89.49)	917 (10.51)		13,421 (89.26)	1572 (10.74)	
Marital status			1500 (<0.001)			2000 (<0.001)			3500 (<0.001)
Never married	11,016 (97.13)	279 (2.87)		13,321 (97.70)	334 (2.30)		24,337 (97.44)	613 (2.56)	
Married	25,771 (83.99)	5203 (16.01)		27,455 (83.12)	5891 (16.88)		53,226 (83.54)	11,094 (16.46)	
Residence			12.70 (<0.001)			1.76 (0.185)			11.39 (<0.001)
Urban	18,846 (87.93)	2667 (12.07)		21,931 (87.05)	3292 (12.95)		40,777 (87.47)	5959 (12.53)	
Rural	17,941 (86.63)	2815 (13.37)		18,845 (87.05)	2933 (12.95)		36,786 (86.85)	5748 (13.15)	
Current Living Children			1600 (<0.001)			2300 (<0.001)			3900 (<0.001)
None	11,578 (96.08)	491 (3.92)		13,607 (96.55)	476 (3.45)		25,185 (96.33)	967 (3.67)	
1–2	15,999 (86.09)	2705 (13.91)		17,568 (85.33)	3179 (14.67)		33,567 (85.68)	5884 (14.32)	
3–5	8368 (80.77)	2009 (19.23)		8886 (79.54)	2333 (20.46)		17,254 (80.14)	4342 (19.86)	
6 or more	842 (74.11)	277 (25.89)		715 (71.74)	237 (28.26)		1557 (73.12)	514 (26.88)	
Socioeconomic									
Employment status			116.20 (<0.001)			156.25 (<0.001)			271.27 (<0.001)
No	16,535 (89.26)	2042 (10.74)		19,059 (88.81)	2385 (11.19)		35,594 (89.02)	4427 (10.98)	
Yes	20,252 (85.77)	3440 (14.23)		21,717 (85.54)	3840 (14.46)		41,969 (85.65)	7280 (14.35)	
Wealth index			14.20 (0.007)			1.95 (0.745) ^a^			8.42 (0.077)
Poorest	8251 (85.38)	1337 (14.62)		8901 (87.53)	1342 (12.47)		17,152 (86.52)	2679 (13.48)	
Poorer	7362 (87.00)	1095 (13.00)		7708 (87.00)	1199 (13.00)		15,070 (87.00)	2294 (13.00)	
Middle	7065 (87.84)	1020 (12.16)		7815 (87.33)	1175 (12.67)		14,880 (87.57)	2195 (12.43)	
Richer	6978 (88.30)	961 (11.70)		8051 (87.17)	1207 (12.83)		15,029 (87.71)	2168 (12.29)	
Richest	7131 (87.60)	1069 (12.40)		8301 (86.36)	1302 (13.64)		15,432 (86.95)	2371 (13.05)	
Health insurance			27.94 (<0.001)			17.77 (<0.001)			46.38 (<0.001)
No	21,863 (88.12)	21,863 (11.88)		15,833 (87.88)	2244 (12.12)		37,696 (88.02)	5295 (11.98)	
Yes	14,924 (85.97)	14,924 (14.03)		24,943 (86.46)	3981 (13.54)		39,867 (86.28)	6412 (13.72)	
Reproductive preference									
Fertility preference			669.81 (<0.001)			942.27 (<0.001)			1600 (<0.001)
Have another	20,195 (90.88)	2129 (9.12)		23,298 (91.16)	2360 (8.84)		43,493 (91,03)	4489 (8.97)	
Undecided	2621 (91.53)	254 (8.47)		1978 (90.85)	209 (9.15)		4599 (91,23)	463 (8.77)	
No more	13,971 (82.39)	3099 (17.61)		15,500 (81.59)	3656 (18.41)		29,471 (81,97)	6755 (18.03)	
Contraceptive			155.55 (<0.001)			385.30 (<0.001)			527.75 (<0.001)
Not Using	20,856 (88.66)	2666 (11.34)		23,731 (89.69)	2868 (10.31)		44,587 (89.20)	5534 (10.80)	
Modern	14,798 (86.22)	2528 (13.78)		15,241 (84.70)	2833 (15.30)		30,039 (85.43)	5361 (14.57)	
Traditional	1133 (79.66)	288 (20.34)		1804 (78.23)	524 (21.77)		2937 (78.76)	812 (21.24)	
Media exposure about family planning									
Radio			2.36 (0.12)			2.92 (0.087)			5.13 (0.024)
No	33,051 (87.36)	4888 (12.64)		37,228 (87.14)	5642 (12.86)		70,279 (87.24)	10,530 (12.76)	
Yes	3736 (86.95)	594 (13.05)		3548 (86.17)	583 (13.83)		7284 (86.57)	1177 (13.43)	
Television			0.66 (0.42) ^a^			3.66 (0.056)			4.19 (0.041)
No	20,166 (87.26)	2973 (12.74)		18,962 (87.24)	2814 (12.76)		39,128 (87.25)	5787 (12.75)	
Yes	16,621 (87.38)	2509 (12.62)		21,814 (86.91)	3411 (13.09)		38,435 (87.11)	5920 (12.89)	
Newspaper			0.52 (0.47) ^a^			3.31 (0.069)			0.50 (0.48) ^a^
No	31,092 (87.39)	4654 (12.61)		35,766 (87.17)	5409 (12.83)		66,858 (87.27)	10,063 (12.73)	
Yes	5695 (86.88)	828 (13.12)		5010 (86.17)	816 (13.83)		10,705 (86.55)	1644 (13.45)	
Others									
Domestic violence			5.49 (0.02)			8.69 (0.003)			13.46 (<0.001)
No	26,275 (87.41)	3831 (12.59)		31,128 (87.24)	4645 (12.76)		57,403 (87.32)	8476 (12.68)	
Yes	10,512 (87.06)	1651 (12.94)		9648 (86.31)	1580 (13.69)		20,160 (86.71)	3231 (13.29)	

n, number of observations; *Χ*^2^, Chi-Square; P, *p* value; IDHS, Indonesian Demographic and Health Survey; ^a^ *p* value ≥.25 not included in the multivariate analysis.

**Table 3:** Factors associated with pregnancy termination among women of reproductive age in Indonesia.

Characteristics	Model 1 (2012 IDHS)		Model 2 (2017 IDHS)		Model 3 (2012 and 2017 IDHS)	
	AOR (95% CI)	*p*	AOR (95% CI)	*p*	AOR (95% CI)	*p*
Demographics						
Age						
15–19	1 (ref.)		1 (ref.)		1 (ref.)	
20–24	1.49 (1.13, 1.95)	0.004	1.57 (1.14, 2.16)	0.006	1.51 (1.23, 1.86)	<0.001
25–29	2.09 (1.60, 2.72)	<0.001	1.94 (1.42, 2.65)	<0.001	2.00 (1.63, 2.44)	<0.001
30–34	3.19 (2.45, 4.15)	<0.001	2.95 (2.16, 4.01)	<0.001	3.03 (2.48, 3.70)	<0.001
35–39	3.83 (2.94, 5.00)	<0.001	3.89 (2.85, 5.30)	<0.001	3.82 (3.13, 4.67)	<0.001
40–44	4.15 (3.17, 5.44)	<0.001	4.48 (3.28, 6.12)	<0.001	4.29 (3.50, 5.26)	<0.001
45–49	4.29 (3.26, 5.64)	<0.001	4.45 (3.25, 6.10)	<0.001	4.34 (3.54, 5.33)	<0.001
Education levels						
No education	1 (ref.)		1 (ref.)		1 (ref.)	
Primary education	1.47 (1.25, 1.73)	<0.001	1.04 (0.86, 1.26)	0.678	1.27 (1.12, 1.44)	<0.001
Secondary education	1.50 (1.26, 1.78)	<0.001	1.07 (0.88, 1.30)	0.493	1.29 (1.14, 1.46)	<0.001
Higher education	1.42 (1.17, 1.72)	<0.001	1.00 (0.81, 1.23)	0.994	1.21 (1.05, 1.39)	0.007
Marital status						
Never married	1 (ref.)		1 (ref.)		1 (ref.)	
Married	4.28 (3.71, 4.93)	<0.001	3.66 (3.20, 4.17)	<0.001	3.95 (3.59, 4.36)	<0.001
Residence						
Urban	1 (ref.)		1 (ref.)		1 (ref.)	
Rural	0.99 (.92, 1.05)	0.675	0.97 (0.91, 1.04)	0.415	0.98 (0.93, 1.03)	0.370
Current Living Children						
None	1 (ref.)		1 (ref.)		1 (ref.)	
1–2	0.76 (0.68, 0.86)	<0.001	0.84 (0.74, 0.94)	0.003	0.80 (0.74, 0.87)	<0.001
3–5	0.86 (0.75, 0.99)	0.025	0.99 (0.87, 1.13)	0.865	0.92 (0.84, 1.01)	0.086
6 or more	1.04 (0.86, 1.26)	0.673	1.14 (0.93, 1.38)	0.200	1.09 (0.95, 1.25)	0.213
Socioeconomic						
Employment status						
No	1 (ref.)		1 (ref.)		1 (ref.)	
Yes	1.04 (0.98, 1.11)	0.200	1.04 (0.98, 1.11)	0.164	1.05 (1.00, 1.09)	0.040
Wealth index						
Poorest	1 (ref.)		–		1 (ref.)	
Poorer	0.96 (0.87, 1.05)	0.327	–	–	1.01 (0.95, 1.08)	0.693
Middle	0.94 (0.86, 1.04)	0.233	–	–	0.98 (0.92, 1.05)	0.592
Richer	0.89 (0.80, 0.98)	0.020	–	–	0.95 (0.89, 1.02)	0.166
Richest	0.98 (0.88, 1.10)	0.777	–	–	1.03 (0.96, 1.12)	0.391
Health insurance						
No	1 (ref.)		1 (ref.)		1 (ref.)	
Yes	1.08 (1.02, 1.15)	0.009	1.06 (0.99, 1.12)	0.074	1.07 (1.02, 1.11)	0.002
Reproductive preference						
Fertility preference						
Have another	1 (ref.)		1 (ref.)		1 (ref.)	
Undecided	0.79 (0.68, 0.91)	0.001	0.84 (0.72, 0.98)	0.026	0.81 (0.73, 0.90)	<0.001
No more	0.90 (0.83, 0.98)	0.011	0.88 (0.82, 0.95)	0.001	0.89 (0.84, 0.94)	<0.001
Contraceptive						
Not Using	1 (ref.)		1 (ref.)		1 (ref.)	
Modern	0.74 (0.70, 0.80)	<0.001	0.78 (0.73, 0.84)	<0.001	0.76 (.72, 0.80)	<0.001
Traditional	0.97 (0.84, 1.12)	0.688	1.13 (1.01, 1.26)	0.031	1.06 (0.98,1.16)	0.152
Media exposure about family planning						
Radio						
No	1 (ref.)		1 (ref.)		1 (ref.)	
Yes	1.11 (1.01, 1.23)	0.029	1.02 (0.93, 1.13)	0.634	1.05 (0.98, 1.12)	0.181
Television						
No	–	–	1 (ref.)		1 (ref.)	
Yes	–	–	1.06 (1.00, 1.13)	0.059	1.10 (1.05, 1.15)	<0.001
Newspaper						
No	–	–	1 (ref.)		–	–
Yes	–	–	1.05 (0.96, 1.15)	0.262	–	–
Others						
Domestic violence						
No	1 (ref.)		1 (ref.)		1 (ref.)	
Yes	1.11 (1.04, 1.19)	0.001	1.14 (1.07, 1.22)	<0.001	1.13 (1.05, 1.15)	<0.001

AOR, adjusted odds ratio; CI, confidence interval; P, *p* value; IDHS, Indonesian Demographic and Health Survey; 1 (ref.), Reference.

## Data Availability

The data presented in this study are available on request from the corresponding author.
